# Hydrogen Sulfide Alleviates Anxiety, Motor, and Cognitive Dysfunctions in Rats with Maternal Hyperhomocysteinemia via Mitigation of Oxidative Stress

**DOI:** 10.3390/biom10070995

**Published:** 2020-07-02

**Authors:** Olga Yakovleva, Ksenia Bogatova, Renata Mukhtarova, Aleksey Yakovlev, Viktoria Shakhmatova, Elena Gerasimova, Guzel Ziyatdinova, Anton Hermann, Guzel Sitdikova

**Affiliations:** 1 Department of Human and Animal physiology, Kazan Federal University, 18 Kremlevskaya str., 420008 Kazan, Russia; a-olay@yandex.ru (O.Y.); kowarik.ru@yandex.ru (K.B.); rena.mukhtarova98@mail.ru (R.M.); alv.yakovlev@gmail.com (A.Y.); vicysic94@mail.ru (V.S.); gerasimova.el.2011@yandex.ru (E.G.); 2 Department of analytical chemistry, Kazan Federal University, 18 Kremlevskaya str., 420008 Kazan, Russia; ziyatdinovag@mail.ru; 3 Department of Biosciences, University of Salzburg, Salzburg 5020, Austria; anton.hermann@sbg.ac.at

**Keywords:** hydrogen sulfide (H_2_S), maternal hyperhomocysteinemia (hHcy), locomotor activity, anxiety, learning and memory, motor dysfunction, H_2_S production, oxidative stress

## Abstract

Hydrogen sulfide (H_2_S) is endogenously produced from sulfur containing amino acids, including homocysteine and exerts neuroprotective effects. An increase of homocysteine during pregnancy impairs fetal growth and development of the offspring due to severe oxidative stress. We analyzed the effects of the H_2_S donor—sodium hydrosulfide (NaHS) administered to female rats with hyperhomocysteinemia (hHcy) on behavioral impairments and levels of oxidative stress of their offspring. Rats born from females fed with control or high methionine diet, with or without H_2_S donor injections were investigated. Rats with maternal hHcy exhibit increased levels of total locomotor activity and anxiety, decreased muscle endurance and motor coordination, abnormalities of fine motor control, as well as reduced spatial memory and learning. Oxidative stress in brain tissues measured by activity of glutathione peroxidases and the level of malondialdehyde was higher in rats with maternal hHcy. Concentrations of H_2_S and the activity and expression of the H_2_S generating enzyme—cystathionine-beta synthase—were lower compared to the control group. Administration of the H_2_S donor to females with hHcy during pregnancy prevented behavioral alterations and oxidative stress of their offspring. The acquisition of behavioral together with biochemical studies will add to our knowledge about homocysteine neurotoxicity and proposes H_2_S as a potential agent for therapy of hHcy associated disorders.

## 1. Introduction

Hydrogen sulfide (H_2_S), established as third gasotransmitter along with nitric oxide and carbon monoxide, is endogenously produced in different tissues and mediates numerous physiological and pathophysiological processes [1,2]. In the central nervous system H_2_S facilitates the induction of long-term potentiation [3], improves fear memory in aged rats [4], modulates neuronal excitability [5,6], increases intracellular Ca^2+^ levels, generates Ca^2+^ waves in astrocytes [7], and is involved in the generation and conduction of somatic and visceral pain [8,9]. Recent studies indicate a neuroprotective role of H_2_S in different neurological pathologies [10,11], including Alzheimer and [12] Parkinson disease [13], epilepsy [14], or cerebral injury after ischemic stroke [15] due to antioxidant features of H_2_S [2,16]. 

Several authors reported that there are four enzymatic pathways involved in the production of H_2_S: cystathionine β-synthase (CBS), cystathionine γ-lyase (CSE), and 3-mercaptopyruvate sulfurtransferase (3MCT) coupled with cysteine amino transferase (CAT) and 3MCT coupled with D-amino acid oxidase (DAO) [2,11,17,18,19]. CBS and CSE are pyridoxal 5′-phosphate (PLP)-dependent enzymes located in the cytosol, whereas PLP-independent 3-MST mainly generates H_2_S within mitochondria. Expression of H_2_S-producing enzymes is tissue specific. CBS and 3-MST are predominantly found in the central nervous system, although these enzymes are also present in peripheral tissues, whereas CSE abundantly occurs in the liver and in vascular and nonvascular smooth muscles [2,11,17,18,19].

Homocysteine produced during the metabolism of methionine [12,20] can be converted back to methionine by remethylation using methionine synthase with 5-methyl-tetrahydrofolate as a methyl group donor. Homocysteine is also metabolized by CBS to cystathionine, which is further metabolized with the help of CSE to cysteine and α-ketobutyrate. Genetic mutations of homocysteine metabolism enzymes, deficiency of B vitamins, excess of methionine, disruption of excretory function and/or certain medications, such as antiepileptic drugs, or L-DOPA, can lead to an increase of homocysteine in the body, called hyperhomocysteinemia (hHcy) [12,20,21,22]. Plasma homocysteine levels ranging from 15 to 30 μM cause mild hHcy, 31 to 100 μM—moderate hHcy or >100 μM—severe hHcy symptoms [23]. An elevated level of homocysteine induces endothelial dysfunctions, oxidative stress, or inflammation and is associated with several pathological states like neurological disorders, chronic kidney disease, osteoporosis, gastrointestinal disorders, cancer, or congenital defects [24,25,26]. Increased homocysteine levels during pregnancy result in preeclampsia, fetus pathologies or growth restriction due to endothelial dysfunctions of the placenta [27]. Prenatal hHcy induces well-known developmental impairments, including delay in reflex maturation or motor and cognitive dysfunctions [28,29,30,31,32,33]. However, little is known about the long-term impact of prenatal hHcy. At the same time population studies indicate that folate insufficiency, high total homocysteine levels and/or low vitamin B12 levels in early pregnancy have long-lasting effects on brain development associated with poor cognitive performance [34].

Elevated levels of homocysteine affect the H_2_S concentration and expression/activity of H_2_S-generating enzymes in vitro and in vivo models [11,20]. Intracerebroventricular injection of homocysteine decreased the expression of CBS and CSE in the rat hippocampus and lead to learning and memory dysfunctions [35,36]. In our previous study H_2_S donor administration to female rats with hHcy during pregnancy restored the developmental impairment of the offspring during the first 3 weeks after birth [28]. The aim of the present study is to evaluate the effects of H_2_S donor on anxiety, motor and cognitive behavior, the level of oxidative stress in brain tissues, concentration of H_2_S, and the activity/expression of CBS in adult rats with maternal hHcy.

## 2. Materials and Methods

### 2.1. Animals

Experiments were carried out using Wistar rats in accordance with EU Directive 2010/63/EU for animal experiments and the Local Ethical committee Kazan Federal University (protocol No. 8 from 5 May 2015). Animals were housed in polypropylene cages (32 × 40 × 18 cm) under controlled temperature (22–24 °C), with a 12:12 L/D light schedule (lights on at 6:00 a.m.) and free access to food and water.

**Figure 1:**
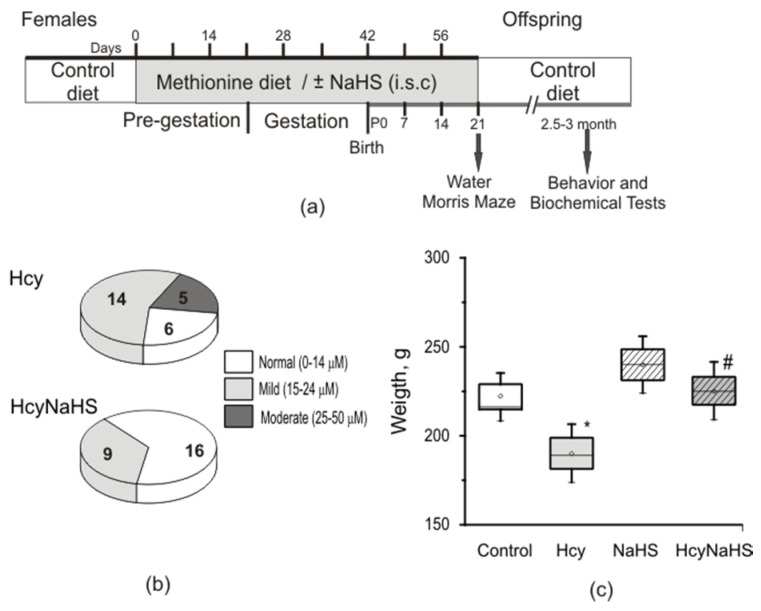
Experimental design and homocysteine level in rats with maternal hHcy. (**a**) Schematic representation of the experimental design. (**b**) Ratio of animals with normal (white sector, 0–14 µM), mild hHcy (grey sector, 15–24 µM), and moderate hHcy (dark grey sector, 25–50 µM) of Hcy and HcyNaHS groups (the number of rats is indicated at the sectors). (**c**) Average weight of rats from control (white box), NaHS (dashed box), Hcy (grey box), HcyNaHS (grey dashed box) groups. Boxes indicate SEM, horizontal lines—median, the small circle inside boxes—mean value, whiskers—5–95 percentile of data. * *p* < 0.05 compared to the control group, ^#^ *p* < 0.05 compared to the Hcy group.

Pregnant rats were divided into four groups as follows: one group was fed ad libitum with a control diet (*n =* 7), the second group (*n* = 11) received daily methionine (7.7 g/kg body weight) with food starting 3 weeks prior to and during pregnancy and lactation until weaning at P21 [29,37]. The third group (*n* = 4) received the H_2_S donor, sodium hydrosulfide (NaHS) three weeks before and throughout pregnancy according the following protocol: 7 days of injections alternated with 7 days of adaptation [28]. Rats of the fourth group (*n* = 4) received daily methionine and injections of NaHS according to the above mentioned protocols (Figure 1a). NaHS was diluted in sterilized saline and injected subcutaneously (i.s.c.) at a dose of 3 mg/kg.

The offspring was divided into four groups in accordance with the diet of the mother: 1) The control diet group (*n* = 25 pups from 7 L); 2) methionine diet group (Hcy *n* = 25 pups from 11 L); 3) control diet group receiving NaHS (NaHS group, *n* = 25 pups from 5 L; 4) methionine diet group receiving NaHS (HcyNaHS group, *n* = 25 pups from 6 L) (Figure 1a).

Behavioral testing, analysis of oxidative stress and H_2_S level, activity and expression of CBS in brain tissues were performed at the age P72–90 with the exception of Morris Water maze test which was performed at P21–22 (Figure 1a). Since no significant differences were registered between male and female rats the data were pooled for subsequent analysis

### 2.2. Behavioral Testing

#### 2.2.1. Open Field

Locomotor activity and anxiety were analyzed using an open field test. Rats were individually placed in a square arena of 100 × 100 cm size with a wall 36 cm high divided into 25 squares of 20 × 20 cm (Open Science, Moscow, Russia) equipped with a video system. The rat was placed into the center of the open field and allowed to explore the apparatus for 3 min with tracking of square crossing, rearing, grooming, defecation, and center zone activity [38]. Each animal was given a score for total locomotor activity calculated as the sum of square crossings and number of rears. After each trial the open field was cleaned with 70% ethyl alcohol and permitted to dry between tests.

#### 2.2.2. Muscle Endurance 

Muscle endurance was assessed by the paw grip endurance (PaGE) test [39]. Rats were placed on a wire grid and gently shaken to prompt the rat to grip the grid. The grid was turned upside down over a housing cage and held at ~0.45 m above an open cage bottom. The time (s) spent on the grid before falling was assessed. The largest value from three individual trials was used for analysis.

#### 2.2.3. The Rotarod Test

The Rotarod test was used to assess the motor coordination of fore and hind limbs and the balance (Neurobotix, Moscow, Russia) [40]. Each rat was placed on the rod with a rotation speed of ten rotations per min (rpm) and the time to fall off and the running distance were measured. Animals are subjected to three consecutive test sessions (trials) with an interval of 20–30 min. The best of the latency to fall off the rotating rod was recorded [41].

#### 2.2.4. Sunflower Seed Task and Vermicelli Handling Test

Sunflower seed task and vermicelli handling test were used to assess the fine motor control. In *sunflower seed task* the ability of animals to use the limb and digits during sunflower seed consumption was estimated [42]. Animals were trained for three consecutive days and on the fourth day the behavior was recorded and videotaped. During the test the rat was placed in a clear plexiglass box (50 × 50 × 50 cm) with five sunflower seeds in the corner of the box. The total amount of time which the rat spent manipulating, opening, and consuming seeds as well as the number of pieces of shell left were recorded. The experimenter started timing the moment the animal touched the first seed and stopped the timer every time the animal was distracted.

In the *vermicelli handling test* rats were given uncooked vermicelli (7 cm lengths and 1.5 mm diameter) and coordinated asymmetrical movement patterns of paws and mouth were observed. Rats most often hold the long piece in both paws and move it into the mouth using a coordinated asymmetrical holding pattern when one paw is used to hold the piece with a whole paw grasp and the other paw to guide the piece into the mouth, often with the digit tips [43]. Atypical behavior patterns were assessed according to previous studies [43,44] and included following patterns: (1) Paws are symmetrical (without a clear separation of functions) when eating long pasta; (2) switching the paws functions from guide to grasp during eating; (3) one of the paws does not contact the pasta during eating, only to adjust the piece of pasta; (4) the piece of pasta is dropped during eating; (5) the mouth is used to pull the pasta piece through the paws; (6) hunched posture, when the rat bends over the pasta piece and moves the mouth down while the piece becomes smaller. The test consisted of three trials with pasta pieces given one at a time per each trial. The rat was placed in a clear plexiglass box and the trials were videotaped for analysis.

#### 2.2.5. Bilateral Tactile Stimulation 

Bilateral tactile stimulation or adhesive removal test was developed to assess movement/sensory asymmetries due to unilateral nigrostriatal damage or stroke-related impairments [45]. The rat was placed in a clear plexiglass box and allowed to habituate for 1 min. Then the rat was picked up and a piece of tape (1 cm long and 3 mm wide) was placed on the ventral side of each paw. The rat was then placed back into the box and allowed to remove each piece of tape using its teeth. Average removal time for each stimulus was calculated using the average across three trials.

#### 2.2.6. Light-Dark Box Test

A light-dark box test was used to assess anxiety of the rat. The light-dark box apparatus (Shelter, Open Science, Moscow, Russia) consists of two equal connected compartments—light and dark. Rodents prefer the dark area and at the same time tend to explore the new environment. These two conflicting emotions lead to observable anxiety like symptoms. The rat was placed in the light compartment and allowed to explore the apparatus for 3 min. Numbers of entries and the time spent in the light compartment were measured [46].

#### 2.2.7. T Maze 

T maze was used to assess spatial working memory in a spontaneous alternation task [47]. The maze is made of non-reflective and odor resistant material equipped with a video-tracking system (Open Science, Moscow, Russia). In a first trial the rat was placed at the starting point (the start arm at the bottom of the “T”) and allowed to explore the right or left goal arm for 3 min. Once the rat entered to a particular goal arm of the “T” junction between the start arm and the opposing goal arm was closed. The rat was left in the maze for 30 s to explore the goal arm, and then placed at the start arm for 30 s before repeating the run. “Alternation” was considered if the rat entered the opposite arm as compared to the previous run. In 10 min breaks two more trials with two runs were performed. Alternation in each trial was counted as 33.3% and in case of selection of the opposite arm in three trials the rat scored 99.9%.

#### 2.2.8. Morris Water Maze 

Morris water maze is a widely used method to study learning and spatial memory [48]. The water maze consists of a round pool (1 m in diameter and 0.4 m deep) filled with water of 26 °C where non-fatty dry milk was added to make the water opaque. The escape platform was placed in one of the sectors of the pool. The rat was allowed to swim and search for the platform for a maximum of 180 s. The animal was carefully lowered into the water. In the beginning the rat was swimming around the edge of the pool looking for a way out. Eventually, the animal learned to find the platform and climbed onto it. The time spent to find the platform and swimming trajectory were estimated using a video tracking system. Learning was assessed by the platform search time (escape latency), swimming speed, and distance during six consecutive trials. To measure spatial memory for the previous platform location the probe trials in which the platform was removed from the pool were performed in 1 h and 24 h after the last learning trial. We assessed the time necessary to reach the target quadrant where the platform was located and the relative number of rats swimming in this area.

The swimming trajectory was analyzed manually and four major types of behaviors were determined: thigmotaxis, where an animal spends most of the time next to the wall; target scanning—scanning of the region around the platform; incursion, where the animal still touches the wall but starts moving inwards and scanning, where the more central regions of the arena are searched [49].

### 2.3. Biochemical Analysis

#### 2.3.1. Measurements of Plasma Homocysteine Level

The total homocysteine level in the plasma was determined by electrochemical detection using nano-carbon modified electrodes as described previously [50,51].

#### 2.3.2. Assay of H_2_S Generation

Total sulfide as relative marker of H_2_S concentration and H_2_S generation assays were carried out using the *N*,*N*-dimethyl-p-phenylenediamine sulphate (NNDPD) method [52]. Brain tissues of rats P72–90 were homogenized in phosphate buffered ice-cold 0.15 M NaCl solution. The homogenate (10%, 860 µL) was mixed with zinc acetate (1%, 500 µL) and 0.15 M NaCl (140 µL) at room temperature. Trichloroacetic acid (10%, 500 µL) was added to stop the reaction and zinc acetate (1%, 500 µL) to trap H_2_S. To assess the rate of H_2_S production the probe homogenate (10%, 860 µL) was mixed with L-cysteine (10 mM, 40 µL), pyridoxal 5′-phosphate (2 mM, 40 µL), and saline (60 µL) and incubated at 37 °C for 60 min. Trichloroacetic acid and zinc acetate were administrated to trap the produced H_2_S. 

In both cases probes were mixed with NNDPD (20 mM, 266 µL) in 7.2 M HCl and FeCl_3_ (30 mM, 266 µL) in 1.2 M HCl and absorbance of aliquots of the resulting solution (600 µL) was measured at 670 nm by spectrophotometry (PE-5300VI, ECOHIM, Saint Petersburg, Russia). Total sulfide concentrations were calculated against a NaHS calibration curve. H_2_S synthesizing activity is expressed as µM H_2_S produced by 1 g tissue per minute (µM/min/g).

#### 2.3.3. Lipid Peroxidation and the Activity of Glutathione Peroxidases

Samples of brain tissue were frozen and homogenized in buffer solution (0.15 M NaCl with phosphate buffer, ratio 1:10) for further analysis. Malondialdehyde (MDA) was measured spectrophotometrically according to the method of Ohkawa et al. 1979 [53]. Homogenates of brain tissue were mixed with 20% trichloroacetic acid and 0.03 М 2-thiobarbituric acid at a ratio 2:2:1. The mixture was heated for 45 min at 95 °C and centrifuged for 10 min at 10,000 g. Under this condition MDA readily participates in a nucleophilic addition reaction with 2-thiobarbituric acid, generating a red, fluorescent 1:2 MDA adduct. The absorbance of the supernatant was monitored at 532 nm (ε_TBA-MDA_ = 1.55 mM^−1^ cm^−1^) by spectrophotometry (PE-5300VI, ECOHIM, Saint Petersburg, Russia). MDA levels were expressed as µg/g of tissues.

The antioxidant potential was determined by measuring the activity of glutathione peroxidase (GPx) assessed by the decrease of the reduced form of glutathione (GSH) level using tert-butyl as a substrate [54]. One mL of glutathione solution was mixed with 1 mL of brain homogenate; the mixture was divided into two centrifuge tubes (test and control) and incubated for 5 min. Tert-butyl hydroperoxide solution (5 μM, 0.02 mL) was added to the test tube. After 10 min, 0.2 mL of cold 10 % trichloroacetic acid was poured into the test and control tubes. Samples were centrifuged for 15 min at 10,000 *g* and 0.1 mL of the supernatant from the control and test tubes were transferred into the chemical tubes and 2 mL of phosphate buffer (pH 8.0) and 0.05 mL of Ellman’s reagent were added and mixed. The optical density of the control and test samples was measured at 412 nm by a spectrophotometer (PE-5300VI, ECOHIM, Saint-Petersburg, Russia). GPx activity was expressed as µg/g of tissues per min.

### 2.4. Western Blot

The cortex, hippocampus, and cerebellum of rats were quickly removed, dropped in ice-cold lysis buffer (20 mM Tris-HCl with pH 7.5, 150 mM NaCl, 1 mM EDTA, 1% Triton) containing a protease inhibitor cocktail tablet (S8820, Sigma-Aldrich, St. Louis, MO, USA) and centrifuged at 12,000 g, at 4 °C for 15 min. The supernatant was mixed with sample buffer (50 mM Tris-HCl, pH 6.8, 10% sodium dodecyl sulfate (SDS), 10% glycerol, 0.01% bromophenol blue, 10% β-mercaptoethanol) and was heated at 95 °C for 5 min and used for Western blotting. Equal amounts of protein from homogenates were separated by 10% SDS-polyacrylamide gel electrophoresis and then transferred onto 0.45 μm polyvinylidene difluoride (PVDF) membranes (Thermo Scientific Pierce, Waltham, MA, USA) with an electro-blotting apparatus (Mini-PROTEAN Tetra cells, Bio-Rad, Hercules, CA, USA). The PVDF membranes were blocked with a solution containing 3% bovine serum albumin (BSA) at room temperature for 60 min, and then reacted with a primary antibody anti-CBS (1:500, sc-271886, Santa Cruz Biotechnology Inc., Dallas, TX, USA). After washing, blots were reacted with horseradish peroxidase-conjugated secondary antibodies (1:3000; ab205719, Abcam, Cambridge, MA, USA) at room temperature for 2 h. Bound antibody was visualized with an ECL chemiluminescence system. Images were captured using ChemiDoc XRS (Bio-Rad, Hercules, CA, USA), and detected bands were quantified with Image Lab Software (Bio-Rad, Hercules, CA, USA). The intensity of each band was normalized to GAPDH expression (1:2500; ab8245, Abcam, Cambridge, MA, USA). Protein content was measured using biuret reaction containing BSA as a standard [55]. The absorbance was assessed by a spectrophotometer at 540 nm. Results were calculated from four repeats. 

### 2.5. Statistical Analysis

Normality of the sample data was evaluated with the Shapiro-Wilk test (sample size less than 25) or Kolmogorov-Smirnov test (sample size more than 25) for equal variances using F-test Origin Pro software (OriginLab Corporation, Northampton, MA, USA). Data are expressed as median (Q1–Q3) or mean ± SEM. Statistical significance between medians was calculated using nonparametric ANOVA Kruskal-Wallis test and Mann-Whitney test in Origin Pro 2015 (OriginLab Corporation, Northampton, MA, USA). Statistical significance between means was calculated using parametric One Way ANOVA or Two Ways ANOVA (for Morris water maze) followed by Bonferroni test or Student’s *t*-tests (two-tailed) in Origin Pro 2015 (OriginLab Corporation, Northampton, MA, USA). Differences were considered as statistically significant at *p* < 0.05; n indicates the number of animals.

## 3. Results

### 3.1. Maternal hHcy Increases Plasma Hcy Level and Decreases the Weight of the Rat Offspring. Effects of H_2_S Donor

It is well-known that maternal hHcy induces growth retardation and delayed development of the offspring during the early postnatal period [28,29,30,31,32]. In our experiments we focused on the effects of maternal hHcy and NaHS administration during pregnancy on the adult offspring with the exception of Morris tests, performed at P21–22. The concentration of plasma homocysteine in control females was 7.9 ± 0.3 µM (*n* = 12) which was increased more than three times in dams fed with methionine-containing diet (27.3 ± 2.4 µM, *n* = 15, *p* < 0.05). The concentration of homocysteine in the plasma of pups born from control females at P90 was 6.3 ± 0.3 µM (*n* = 22). In adult rats with prenatal hHcy about 76% of animals had elevated levels of homocysteine with mild (56%, *n* = 14, Figure 1b) or moderate (20%, *n* = 5, Figure 1b) levels; on average 19.5 ± 1.5 µM (*n* = 25, *p* < 0.05). NaHS treatment did not change homocysteine levels in control dams (8.3 ± 0.3 µM, *n* =10) and their pups (6.4 ± 0.4 µM, *n* = 25). However, it significantly reduced the homocysteine concentration in dams with hHcy (14.5 ± 1.5 µM, *n* = 8) and their adult offspring. 64% of rats from the HcyNaHS group showed normal and 36% (*n =* 9, Figure 1b) a mild increase of homocysteine concentrations, which on average was 12.1 ± 1.2 µM (*n =* 25, *p* < 0.05). The weight of P90 rats from the Hcy group was significantly lower compared to controls and HcyNaHS groups (Figure 1c).

### 3.2. Effects of Maternal hHcy and NaHS on Behavior in the Open Field Test

**Figure 2:**
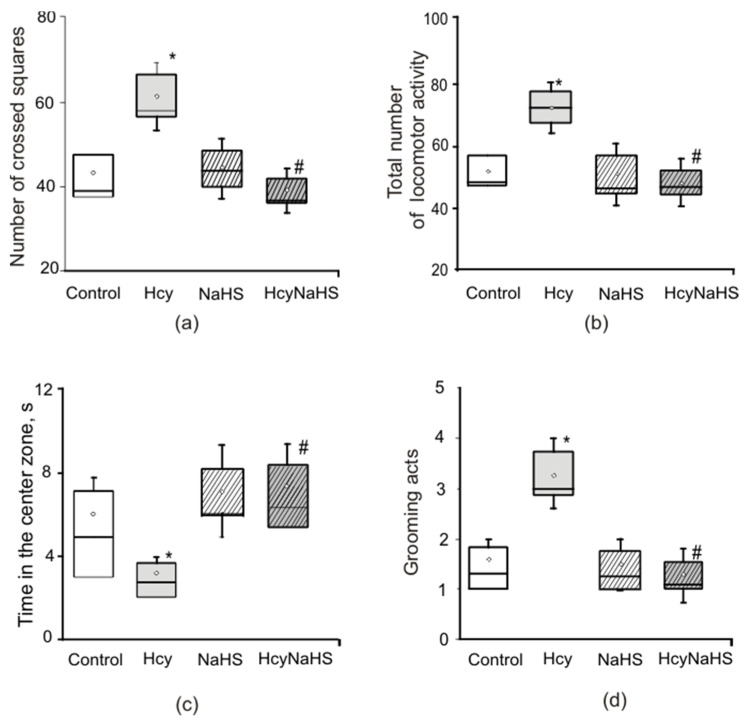
Effects of maternal hHcy and NaHS treatment on behavior in the open field test. Number of crossed squares (**a**), total locomotor activity (**b**), time spent in the central square (**c**), and grooming (**d**) of rats compared to control (white boxes), Hcy (grey boxes), NaHS (dashed boxes), HcyNaHS (grey dashed boxes) groups. * *p* < 0.05 compared to the control group, **^#^** *p* < 0.05 compared to the Hcy group.

Locomotor and exploratory activity was assessed in the open field test. Horizontal activity (square crossing) was significantly higher in the Hcy group compared to control and NaHS groups (Figure 2a). The number of crossed squares of rats from the NaHS group was not different from the control group (Figure 2a). Rearing or vertical activity of rats were not different in all groups (8.7 ± 1.2 in control; 11.1 ± 0.6 in Hcy; 8.4 ± 0.9 in NaHS; 9.6 ± 1.1 in HcyNaHS groups), however, the total locomotor activity was significantly higher in the Hcy group compared to control and NaHS groups (Figure 2b). 

Grooming behavior, the time spent in the center square, center square entries and defecation scores reflect the emotionality of animals [41]. No significant intergroup differences were found in scores of defecation (0.8 ± 0.3 in control; 1.1 ± 0.2 in Hcy; 0.9 ± 0.3 in NaHS; 1.1 ± 0.3 in HcyNaHS groups) and center square entries (6.1 ± 1.1 in control; 3.1 ± 0.4 in Hcy; 7.1 ± 1.1 in NaHS; 7.5 ± 1.7 in HcyNaHS groups). However, rats from the Hcy group spent less time in the central square (Figure 2c) and showed more grooming episodes (Figure 2d). NaHS treatment restored these parameters to control values (Figure 2).

### 3.3. NaHS Decreases the Anxiety Level Measured in the Light-Dark Box in Rats with Maternal hHcy 

**Figure 3:**
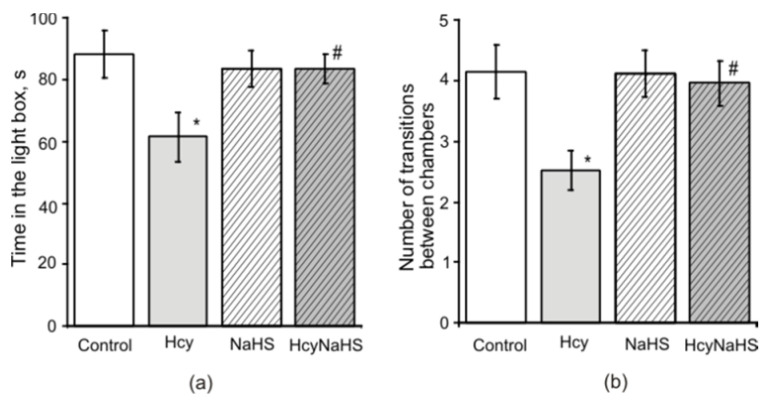
Effects of prenatal hHcy and NaHS treatment on anxiety measured in the light-dark box. The time spent in the light box (**a**) and numbers of transition between chambers (**b**) of rats compared to control (white columns), Hcy (grey columns), NaHS (dashed columns), HcyNaHS (grey dashed columns) groups. * *p* < 0.05 compared to the control group, **^#^** *p* < 0.05 compared to the Hcy group.

The light dark box test creates a conflict situation for an animal that tends to explore an unfamiliar area and the time spent in the dark compartment correlates with the level of anxiety [46]. In the control group time spent in the light compartment was 88.4 ± 7.1 s (*n =* 25) and significantly decreased in the Hcy group (62.5 ± 8.4 s, *n =* 25, Figure 3a). In NaHS and HcyNaHS groups this parameter was not significantly different from the control group. Similar results were obtained for the number of transitions between chambers (Figure 3b).

### 3.4. NaHS Increases Muscle Endurance and Motor Coordination of Rats with Maternal hHcy

**Figure 4:**
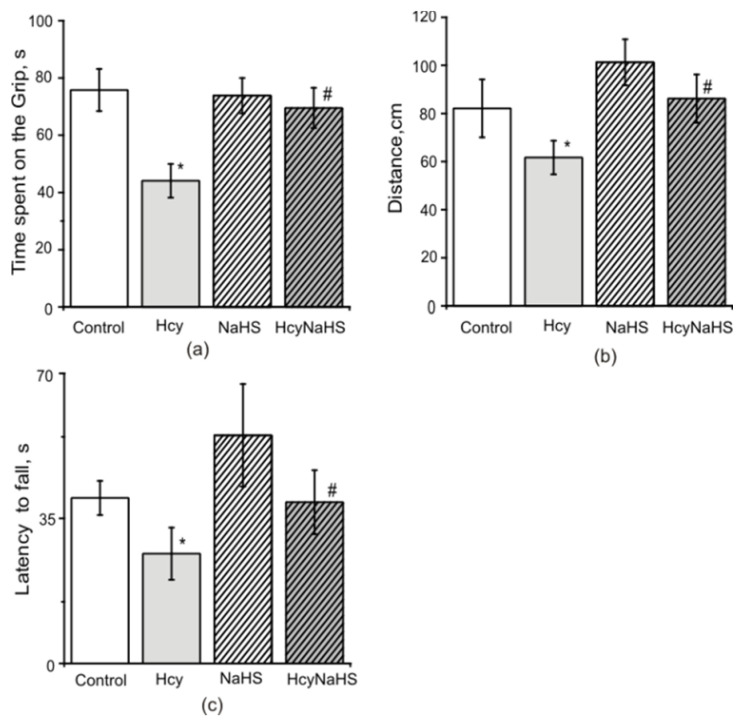
Effects of maternal hHcy and NaHS treatment on muscle endurance and motor coordination. The time spent on the grid (before falling) in paw grip endurance (PaGE) test (**a**), distance (**b**) and latency to fall (**c**) in Rotarod test of rats compared to control (white columns), Hcy (grey columns), NaHS (dashed columns), HcyNaHS (grey dashed columns) groups. * *p* < 0.05 compared to the control group, ^#^ *p* < 0.05 compared to the Hcy group.

In the paw grip endurance (PaGE) test control rats were able to stay on the grid for 75.8 ± 7.3 s (Figure 4a). In the Hcy group this time was significantly lower compared to the control group (44.1 ± 5.8 s, *p* < 0.05). In NaHS and HcyNaHS groups this parameter was not different to the control (Figure 4a). Motor coordination was assessed in Rotarod tests, where the time to fall off and running distance were measured [41]. Similar changes were observed in Hcy group for the Rotarod distance during experimental sessions (Figure 4b). A significant reduction of time spent on the Rotarod was observed in the Hcy group compared to the control group (Figure 4c). NaHS treatment restored both to control values (Figure 4b,c).

### 3.5. Fine Motor Control and Sensory Motor Asymmetry in Rats with Maternal hHcy. Effects of NaHS 

**Figure 5:**
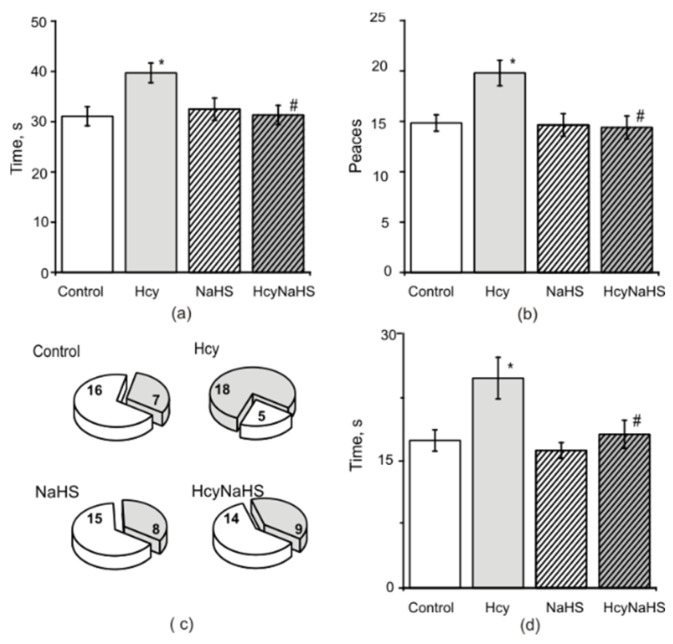
Effects of maternal hHcy and NaHS treatment on fine motor control. Average time to open and successfully consume five sunflower seeds (**a**) and the number of broken shell pieces (**b**) after consumption in the sunflower seed test. The ratio of atypical (grey sector) and typical (white sector) behavior patterns in the pasta handling test in control; Hcy, NaHS, and HcyNaHS groups (number of rats is indicated at the sectors) (**c**). The removal time in the adhesive test of rat from control (white columns), Hcy (grey columns), NaHS (dashed columns), HcyNaHS (grey dashed columns) groups (**d**). Data are expressed as mean ± SEM. * *p* < 0.05 compared to the control group, ^#^ *p* < 0.05 compared to the Hcy group.

*In Sunflower seed tests* rats of the control group quickly learned how to open and consume the sunflower seeds. The average time to open and successfully consume the five sunflower seeds was 31.1 ± 1.8 s and the average number of broken shell pieces after consumption was 14.8 ± 0.8 (Figure 5a,b). In the Hcy group the time of seed consumption and the total number of pieces increased significantly (Figure 5a,b). Both parameters in NaHS and HcyNaHS groups were not different from the control group (Figure 5a,b).

*In the Pasta Handling test* rats most of the time start to eat pasta using a coordinated asymmetrical holding pattern where one paw is mainly used to grasp and the other to guide a piece of pasta to the mouth [43,44]. In the control group 93% of rats used this pattern and only 7% of rats held a piece of paste symmetrically between their paws. Asymmetrical holding pattern at the onset of eating was observed only in 24% of rats from the Hcy group and other rats did not show a clear holding pattern. Rats from the control group showed more adjustment movements compared to the Hcy group. Most rats from the control group used either the right paw (*n =* 15) or the left paw (*n =* 10) to capture the pasta. At the same time, when eating pasta, a change of paws was not observed in animals of the control group, while in animals of the Hcy group, the change of “capture” and “direction” paws was observed in 32% of the trials. Among the atypical/sporadic behaviors that were quantified in control rats, the most common were the “paws together” for long piece of pasta (*n =* 3), the fall of pasta, and a hunched posture (*n =* 2). In animals from the Hcy group atypical behavior additionally included lack of contact to pasta, improper paw position with short and long pasta, and mouth pulling (see Materials and methods). In general, atypical behavior was observed in 30% of rats (*n =* 7) from the control group and 78% of rats (*n =* 18) from Hcy group (Figure 5c). In NaHS and HcyNaHS groups atypical behavior was demonstrated in 35% (*n =* 8) and 40% (*n =* 9) of rats, respectively (Figure 5c). 

In the *adhesive removal tests* sensorimotor functions and sensory asymmetries can be measured [45]. In the control group the average time to remove each piece of tape was 15.2 ± 1.3 s (Figure 5d). In the Hcy group the removal time increased significantly to 21.7 ± 2.1 s (*p* < 0.05, Figure 5d). The mean time to remove each piece for NaHS and HcyNaHS groups was the same as in the control group (Figure 5d). No sensory asymmetry during the test in control and Hcy groups was observed.

### 3.6. NaHS Improves Spatial Learning and Memory of Rats with Maternal hHcy 

**Figure 6:**
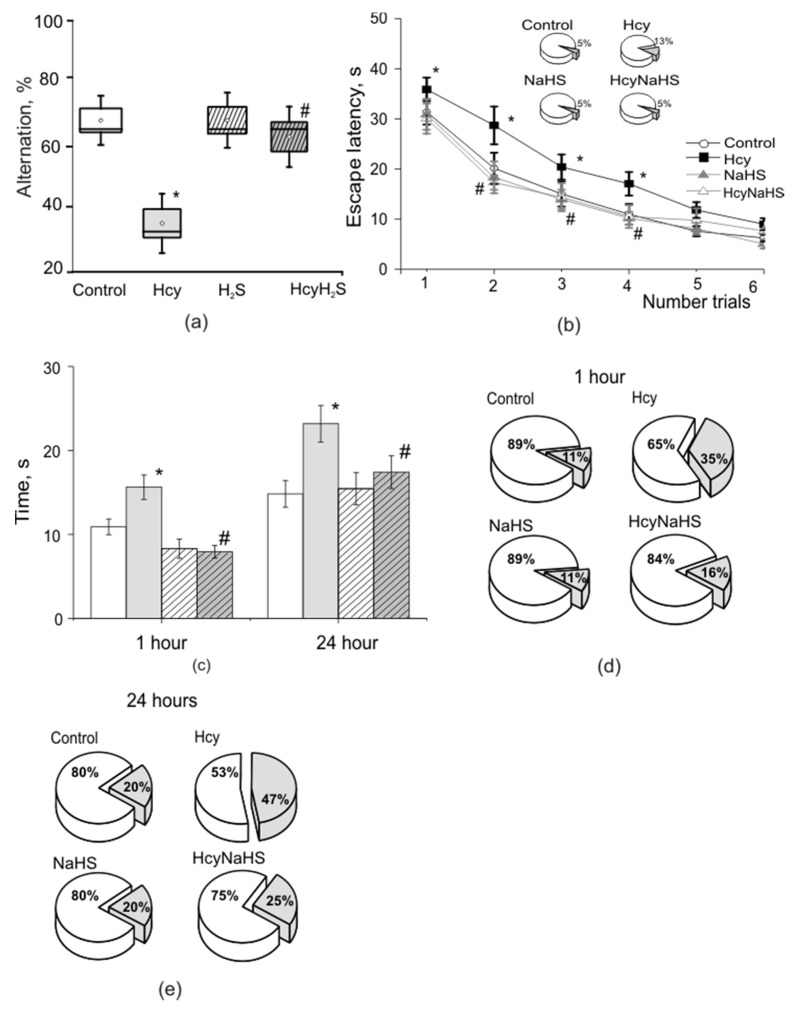
Effects of maternal hHcy and NaHS treatment on cognitive functions in T maze and Morris test. (**a**) Percentage of alternation in the T maze of rats from control (white box); Hcy (grey box), NaHS (dashed box), HcyNaHS (grey dashed box) groups. (**b**) Escape time during learning in the Morris maze of rats in control (white circles), Hcy (grey circles), NaHS (white square), HcyNaHS (grey square) groups. The insert indicates the percentage of rats able of learning (white sector) and failures (grey sector). (**с**) Time necessary to find the target quadrant in 1 h and 24 h after the training of rats from control (white columns), Hcy (grey columns), NaHS (dashed columns), HcyNaHS (grey dashed columns) groups. Ratio of animals able to remind the platform location (white sectors of the circles) and failures (grey sector) after 1 h (**d**) and 24 h (**e**). * *p* < 0.05 compared to the control group, ^#^ *p* < 0.05 compared to the Hcy group.

*T maze* is commonly used for assessing spatial working memory in rats and mice, especially for delayed alternation tasks [47]. In the control group, the average percentage of alternation of the maze arm was 69.2 ± 3.7% (Figure 6a). Rats from the Hcy group showed less alternation—36.3 ± 4.4%, and results obtained from NaHS and HcyNaHS groups were not different to the control group—69.2 ± 4.3% and 64.2 ± 4.8%, respectively (Figure 6a).

In the *Morris water maze* test the time necessary to find the hidden platform (escape time) and distance to find the platform decreased from the first to sixth trial in all groups of animals (Figure 6b, Figure S1; Table S1). In the control group the escape time reduced from 30.5 ± 1.8 s during the first trial to 6.2 ± 0.6 s during the sixth trial (*p* < 0.05, Figure 6b, Table S1). The distance travelled significantly reduced from 201.8 ± 25.7 cm during the first trial to 44.2 ± 5.3 cm during the sixth trial, *p* < 0.05), while the swimming speed remained constant (7.2 ± 0.9 cm/s versus 7.0 ± 1.2 cm/s, *p* > 0.5) (Table S1). The analysis of platform search strategy demonstrated that thigmotaxis and scanning were prevailing during the first two trials substituted by target scanning as a main strategy by the end of the training (4–6 trials) which is the most effective in finding the platform (Figure S1).

The initial escape time was longer for rats from the Hcy group (39.2 ± 1.8 s, *p* < 0.05) compared to controls and decreased to 9.0 ± 1.1 s (*p* < 0.05) during the sixth trial (Figure 6b, Table S1). Significant longer travel distances were observed for rats from the Hcy group during the first (299.4 ± 18.1 cm, *p* < 0.05) and sixth (65.1 ± 10.1 cm, *p* < 0.05) trial compared to the control group. The swimming speed did not differ significantly from control: 7.0 ± 0.6 cm/s, during the first and 7.2 ± 1.0 cm/s and the sixth trial (Table S1). Main searching strategies included thigmotaxis and incursion associated with low chances of finding the platform and therefore increased escape time (Figure S1). Even toward the end of the training thigmotaxis strategy predominated in rats from the Hcy group (Figure S1). Moreover, less animal from the Hcy group (87%) could learn to find the hidden platform compared to the control group (95%) (Figure 6b insert). NaHS administration significantly decreased the escape latency of rats with prenatal hHcy (Figure 6b). In NaHS and HcyNaHS groups the percentage of animals able to learn was 95% (Figure 6b insert)—similar to the controls. The length of swimming trajectories, escape time, swimming speed, and searching strategies of rats obtaining NaHS and HcyNaHS groups were not different from the control group (Figure 6b, Figure S1, Table S1). It is interesting to note that in rats of the NaHS group scanning strategy prevailed even at the beginning of learning. In the HcyNaHS group three types of searching were observed: in the beginning of the training (thigmotaxis, incursion, and target scanning) followed by only scanning at the end of the training (Table S1).

Next we assessed the trajectory and the time necessary for rats to find the target quadrant where the platform was located 1 h and 24 h after the training. Within one hour after the sixth learning trial 89% of the rats from the control group could find the target sector in 11.0 ± 0.9 s with a trajectory length of 63.3 ± 6.1 cm at a constant speed of 5.8 ± 0.5 cm/s, *p* > 0.05) (Figure 6c,d; Table S1). Only 65% of rats from the Hcy group could find the target quadrant. The time necessary to reach the target quadrant (15.3 ± 1.1 s) and the trajectory were significantly longer (91.9 ± 13.1 cm, *р* < 0.05) compared to controls, although the swimming speed was not different (5.8 ± 0.3 cm/s, *р* > 0.05) compared to the control group (Figure 6c,d; Table S1). Similar data were obtained for the 24-h trial (Figure 6c,e; Table S1). Only 53% of rats from the Hcy group were able to find the target sector in about 23 s, which was significantly longer compared to the control group with 15.4 s for 80% of rats (Figure 6d,e; Table S1).

Analysis of searching trajectories indicates that scanning and target scanning strategies were used by animals from the control group. Rats from the Hcy group were swimming along the walls and used more simple exploration strategies such as incursion and thigmotaxis. Searching strategies, the time necessary to reach the target quadrant and trajectory length of NaHS and HcyNaHS groups did not differ from the control group (Figure 6c–e, Figure S1; Table S1).

### 3.7. Effects of Maternal hHcy and NaHS Administration on the Level of Oxidative Stress, H_2_S Concentration, CBS Activity and Expression in Brain Tissues 

#### 3.7.1. H_2_S Level

**Figure 7:**
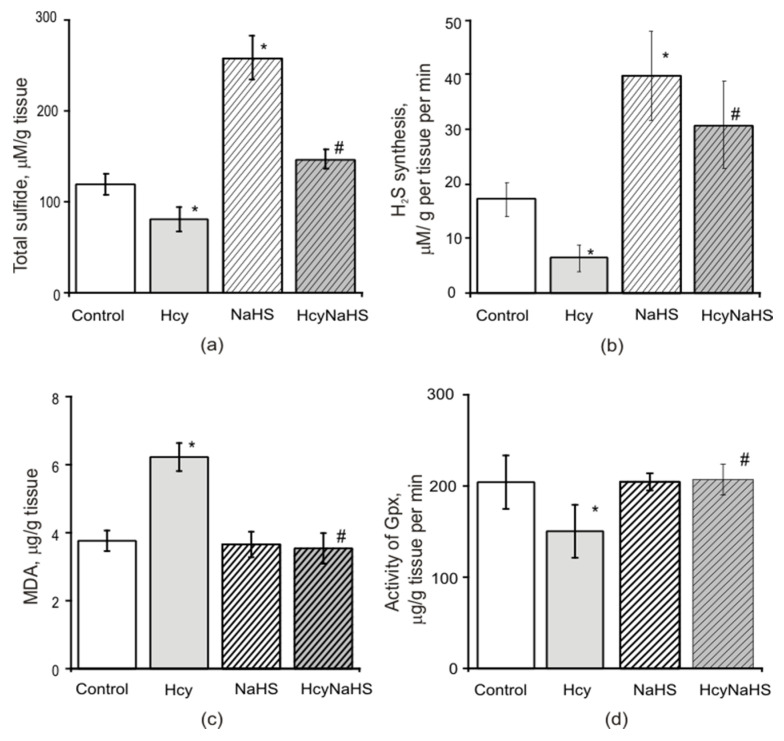
Effects of maternal hHcy and NaHS treatment on H_2_S concentration and the level of oxidative stress in rat brain tissues. H_2_S concentration (**a**) and activity of CBS (**b**) of rat brain tissues from control (*n =* 21), Hcy (*n =* 21), NaHS (*n =* 21), and HcyNaHS (*n =* 21) groups. The level of malondialdehyde (MDA) an end product of lipoperoxidation (**c**) and activity of the antioxidant enzyme—glutathione peroxidase (GPx) (**d**) in brain tissues of rats control (*n =* 20, white columns); Hcy (*n =* 20, grey columns), NaHS (*n =* 19, dashed columns), HcyNaHS (*n =* 20, grey dashed columns) groups. * *p* < 0.05 compared to the control group, ^#^ *p* < 0.05 compared to the Hcy group.

It was shown previously that exposure to homocysteine decreased the endogenous generation of H_2_S [35,56,57]. In our experiments the H_2_S concentration measured as total sulfide level in brain tissues of control animals was 120.1 ± 11.4 µM/g tissue (*n =* 21) (Figure 7a). In rats from the Hcy group we observed a decrease of the H_2_S concentration to 80.3 ± 13.3 µM/g tissue (*n =* 20, *p* < 0.05), which was elevated by NaHS administration in the HcyNaHS group to 147.1 ± 10.5 µM/g tissue (*n =* 20, *p* < 0.05) and the H_2_S group to 259.1 ± 48.2 µM/g tissue (*n =* 12) (Figure 7a). The activity of H_2_S producing enzymes was measured by the rate of endogenous H_2_S generation after high concentrations of cysteine and pyridoxal-5-phospate were added to brain homogenates (Figure 7b). The rate of H_2_S production decreased from 17.1 ± 3.1 µM/min/g in control (*n =* 20) to 6.4 ± 2.4 µM/min/g in the Hcy group (*n =* 20, *p* < 0.05). In the NaHS group the rate of H_2_S production increased up to 39.7 ± 8.1 µM/min/g (*n =* 12, *p* < 0.05) and in the HcyNaHS group—up to 30.7 ± 7.9 µM/min/g (*n =* 20, *p* < 0.05) (Figure 7b).

#### 3.7.2. Lipid Peroxidation and Activity of Glutathione Peroxidase

In order to estimate the extent of oxidative stress in rats with prenatal hHcy the level of malondialdehyde (MDA) was measured in brain tissues of P72–90 animals from control, Hcy, NaHS, and HcyNaHS groups. The MDA level increased almost twice in the Hcy group indicating lipid peroxidation due to production of reactive oxygen species (ROS) (Figure 7c). In rats from HcyNaHS and NaHS groups the MDA level was significantly lower and did not differ from the control group (Figure 7c). Glutathione peroxidases (GPx) activity that reduces peroxides was decreased in the Hcy group of animals and NaHS treatment restored its activity to control values (Figure 7d).

#### 3.7.3. Expression of Cystathionine-Beta Synthase (CBS)

**Figure 8:**
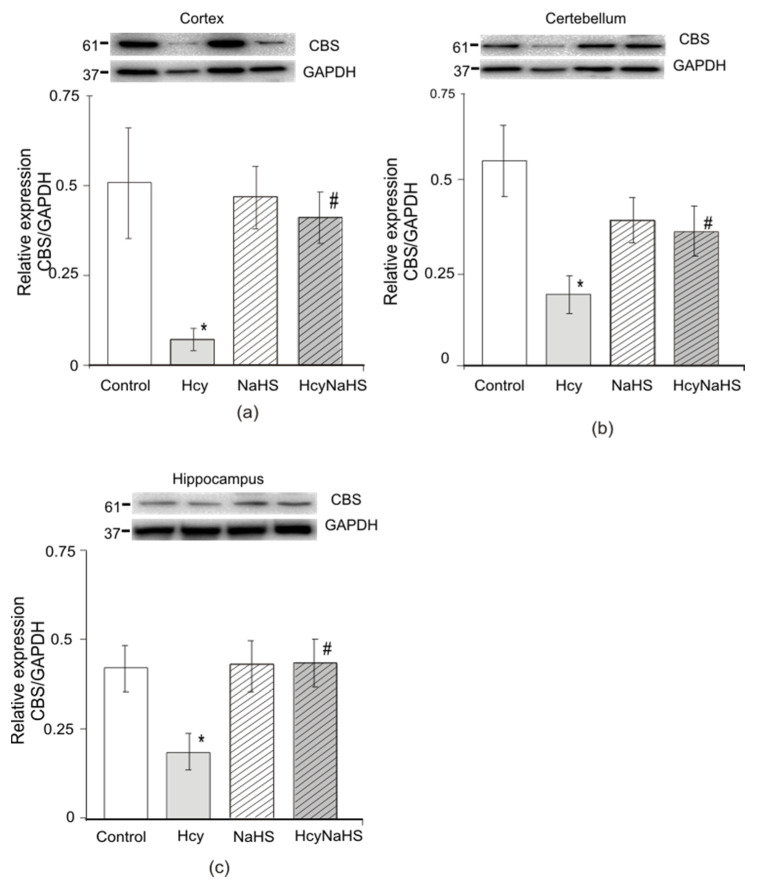
Effects of NaHS administration on the expression of CBS in cortex, cerebellum, and hippocampus of rats with maternal hHcy. Representative Western blot images for CBS and GAPDH proteins in different groups of rats (upper panel) and its quantification (bottom panel) in cortex (**a**), cerebellum (**b**), and hippocampus (**c**) of rats from control, Hcy, NaHS, and HcyNaHS groups after normalization to housekeeping protein GAPDH. * *p* < 0.05 compared to control, ^#^ *p* < 0.05 compared to the Hcy group.

Impaired activity and reduced protein expression of the H_2_S generating enzyme CBS may contribute to the decrease of H_2_S concentration in the brain [58]. We measured the protein level of CBS in cortex, cerebellum, and hippocampus of animals from four experimental groups. Western blot analysis showed that the expression of CBS was significantly reduced in all examined areas in rats from the Hcy group (Figure 8a–c, *p* < 0.05). The level of CBS in the cortex was seven-fold lower in the Hcy group (*n =* 4) compared to the control group (*n =* 3). A similar decrease was found in the hippocampus (~2 fold) and cerebellum (~3 fold) (*n =* 4). CBS expression in brain of rats from NaHS (*n =* 5) and HcyNaHS (*n =* 4) groups was not significantly different from control (Figure 8a–c).

## 4. Discussion

A main finding of our study is that H_2_S exerts a protective action against behavioral abnormalities of rats with maternal hyperhomocysteinemia (hHcy) in late postnatal life because of mitigating oxidative stress and restoration of CBS activity/expression and H_2_S brain level. Our results indicate a neuroprotective role of H_2_S during in-utero development of the fetus under exposure to high homocysteine concentrations.

hHcy during pregnancy induces well-known pregnancy complications followed by developmental impairments of the offspring [22,29,30,31,32,33]. Our previous study demonstrated that homocysteine-evoked oxidative stress during the prenatal period caused delayed brain maturation of the offspring during the first 4 weeks of life [28]. Less attention was paid to late postnatal effects of maternal hHcy, although population studies indicated cognitive dysfunctions of adolescents born with mild hHcy [34]. Therefore, we assessed various behavioral activities such as locomotor activity, anxiety, motor coordination and muscle strength, and working memory in adult rats (P72–90) with maternal hHcy. Spatial leaning and long-term memory was studied in juvenile rats at the age P21–22. In addition, we investigated if the adult offspring with prenatal hHcy display elevated homocysteine levels and oxidative stress. Finally, we administered the H_2_S donor, NaHS, to hHcy females in order to restore the obtained abnormalities.

Despite the fact that the rats were at standard diet after the end of weaning (3 weeks after birth) even at the age of 2.5–3 month we still observed mild (56%) to moderate (20%) hHcy in 76% of the adult offspring. The weight of rats from Hcy group was reduced after birth and did not achieve control values at the age of 2.5–3 month. In open field tests an increased square crossings and grooming along with decreased activity in the central part were observed in rats with prenatal hHcy, indicating an anxiety-phobic behavior reflecting the natural conflict in rats between the tendency to explore a novel environment and the tendency to avoid a bright open area [59]. In a light-dark box the time spent in the light compartment and the number of transitions between chambers was decreased which supports the notion of a higher anxiety level in rats with prenatal hHcy. Increased anxiety may result from decreased S-adenosylmethionine-dependent synthesis of catecholamines due to an excess of homocysteine [25]. Indeed, reduced biosynthesis of dopamine, serotonin, and noradrenalin along with increased activity of the monoamine oxidases–MAO-A and MAO-B in the cerebral cortex and hippocampus was shown in rats with prenatal and postnatal hHcy [60,61].

Motor coordination of limbs was assessed by classic Rotarod and paw grip endurance tests [41]. Our previous study showed a decrease of time spent on a rotating cylinder or on a grid of rats with maternal hHcy during first month of life. In the present study these parameters in adult animals gave similar results, which confirm our opinion of cerebral insufficiency in animals with elevated homocysteine levels. These impairments may result from apoptosis of cerebellar neurons in the early postnatal period due to homocysteine accumulation [31,62]. Moreover, the expression of GluN2C and GluN2D subunits of NMDA receptors mediates a high sensitivity of cerebellar neurons to homocysteine [63].

The delicate manipulations of the forelimbs important for the normal life of rodents were analyzed using the sunflower seed task, vermicelli handling test, and the adhesive test, indicative of sensory and motor abilities of the forelimbs, tongue, and jaw [42,43,44,45]. In hHcy rats impaired manipulation of small objects (seeds, vermicelli) and slowing of the manipulation rate (adhesive test and seed task) was observed which may result from dysfunctions of cortex and subcortical areas responsible for the fine motor control and sensory feedback as also observed in Parkinson disease, cerebral ischemia, or craniocerebral injury [44,64,65]. Indeed, homocysteine is an independent risk factor of Parkinson disease with loss of motor control and L-DOPA treatment leads to elevation of the homocysteine level which aggravates dopaminergic neurodegeneration [66].

Reduced muscle strength and endurance can be explained by skeletal muscle dysfunctions resulting from oxidative/endoplasmic reticulum stress and inflammation observed in hHcy conditions [67]. In addition, impaired synaptic transmission appears to impact skeletal muscle dysfunctions. In fact, less probability of transmitter release and higher sensitivity to inhibitory effects of peroxide was shown in neuromuscular synapses of rats with prenatal hHcy [68]. Likewise, homocysteine amplified the inhibitory effects of hydrogen peroxide on spontaneous acetylcholine release in mouse neuromuscular junction through activation of NMDA receptors [69]. Impaired synaptic transmission in peripheral synapses followed by reduced muscle strength may also underlie the higher risk of amyotrophic lateral sclerosis (ALS) during hHCY [70].

A number of authors report about altered spatial navigation, short-term and long-term memory of rats and mice with pre- and postnatal hHcy [31,32,33,57,60,71,72,73,74]. In our study we found a significant violation of learning and memory of Hcy rats in T maze and Morris water tests where the hippocampus plays a major role [48,75]. In the T maze spontaneous alternation is used to assess working memory based on the innate motivation of the animal to explore its environment [75]. In rats from the Hcy group the ratio of alternations was lower compared to controls alluding to a deficit of short-term memory. This interpretation is supported by results obtained in the Morris water maze test. Rats from the Hcy group needed more time to find the platform during learning and retention trials indicating a deficit of learning and spatial memory. Analysis of explorative strategies used to find the platform has shown that rats from the control group improve their search strategy from a random search (thigmotaxis) during the first trials to scanning. Rats from the Hcy group did not change their strategy of thigmotaxis indicating that their capacity for spatial learning was limited. In probe trials when the platform was removed only 65% of rats from the Hcy group could find the target quadrant within one hour (89% in control) and 53% in 24 h (80% in control) after the learning procedure. These data point to a poor memory consolidation and retention in rats with prenatal hHcy.

Oxidative tissue damage and inflammation in brain tissues play a significant role in homocysteine-induced neurotoxicity [76,77]. Indeed, oxidative stress during the prenatal period markedly reduces spatial learning and memory retention due to disturbed basal transmission in CA3–CA1 synapses and a decrease in hippocampal long-term synaptic potentiation [48,78]. Moreover, homocysteine accumulation during the prenatal period in different brain region including cerebellum, hippocampus, striatum induces excitotoxicity followed by apoptosis [31,79,80] and may impair neuronal network maturation during a critical period of development. This suggestion was supported by results obtained in hippocampal slices of neonatal rats with prenatal hHcy where an increase of neuronal excitability along with a decrease of giant depolarizing potentials important for the establishment of interneuronal connections were observed [81].

Recently, a significant role of H_2_S deficiency in homocysteine-induced neurotoxicity was suggested [11,28,82]. In our study along with increased levels of MDA and decreased activity of GPx, a reduced level of H_2_S and of H_2_S producing enzymes was shown in the brain tissues of adult rats with prenatal hHcy. These findings indicate that maternal hHcy induces long-term alteration of cellular functions in offspring tissues controlling their antioxidant capacity. Apparently, along with detrimental effects of chronic hHcy these changes are mediated at the level of gene expression during the prenatal period because of DNA hyper/hypomethylation. The work of several previous authors indicates that elevated homocysteine levels alter global DNA methylation and induces promoter specific methylation in many genes implicated in atherosclerosis, osteoporosis and other disorders [83,84].

In chronic models of hHcy a decreased expression of CBS and CSE, responsible for endogenous H_2_S generation along with reduced plasma level of H_2_S was shown [35,36,74,82,85]. In high methionine diet mice global methylation and DNA methyltransferase-1 (DNMT1) expression were increased and hyper-methylation of the CBS promoter downregulated CBS expression in bone marrow-derived endothelial progenitor cells [84]. These epigenetic mechanisms may interfere with the gene expression during the early prenatal period the consequences of which are manifested in postnatal life. Indeed, prenatal stress and hypoxia in critical periods of brain development via genetic and epigenetic mechanisms result in significant changes of cognitive functions and increases the risk of neurodegeneration in later life [86,87,88].

DNA hyper-methylation may explain the decrease of CBS expression/activity and low H_2_S level in Hcy rats associated with numerous behavioral alterations as demonstrated in our present study. CBS expression and H_2_S synthesis are important for both—early brain development and support of synaptic plasticity in adult life. The expression of CBS in the nervous system increases from the late embryonic to the early postnatal period [89,90] and plays a major role in the decrease of the homocysteine level and H_2_S synthesis. At early stages of brain development hyperexcitability of neurons increases the probability of seizure-like events [91]. At the same time H_2_S decreases the excitability of the hippocampus during the neonatal period due to an inhibitory action on GluN1/2B NMDA receptors supporting its important role in the inhibitory/excitatory balance of the developing brain [92]. This inhibitory action of H_2_S counteracts the homocysteine induced hyperexcitability. On the other hand, activation of the synaptic GluN2A NMDA receptors by H_2_S supports synaptic plasticity and induction of LTP [3] and appears to exert a protective role in the cognitive decline during aging and neurodegenerative disorders [6].

In our study H_2_S donor injections in hHcy dams not only counteracted oxidative stress and behavioral dysfunctions but also increased CBS expression in brain tissue of the adult offspring proposing an epigenetic control of gene expression during the prenatal period. Similarly, CBS and CSE expression was restored by NaHS injection into the hippocampus of hHcy mice [36]. Moreover, NaHS downregulated CSE but upregulated CBS in cardiomyocytes [93]. Anti-oxidant effects of H_2_S are mediated by direct scavenging of free radicals, including hydrogen peroxide, peroxynitrite, lipid hydroperoxides, and hypochlorous acid [94] or by increasing the activity of endogenous anti-oxidants, such as the glutathione system, superoxide dismutase, or catalase [95]. In addition, H_2_S activates the nuclear-related factor erthyroid 2 [Nrf2] pathway, which is supposed to be a key regulator in cell defense against oxidative stress [57,96,97].

Furthermore, H_2_S plays an important role in the regulation of the placenta vascular tone through an increase of nitric oxide production and more directly by activation of smooth muscle ATP-dependent K^+^ channels [98]. In the stem villus artery CSE loss is associated with human intra-uterine growth restrictions [99]. The uterine artery CBS expression increases during pregnancy, whereas decreased maternal serum sulfide and reduced placental CBS and CSE correlate with preeclampsia [98,100]. These findings can explain the positive effects of H_2_S donor treatment during pregnancy on the offspring development.

Several studies revealed cellular mechanisms of neuroprotective effects of H_2_S in homocysteine induced neurotoxicity in a postnatal model of hHcy [35,36,57] which are also applicable to our model of hHcy. H_2_S prevented the increased anxiety of hHcy rats due to elevation of the catecholamines level [61]. In chronic unpredictable mild stress, H_2_S protected hippocampal damage through activation of the BDNF-TrkB (brain-derived neurotrophic factor—tyrosine protein kinase B) pathway and inhibition of hippocampal CA1 long-term depression due to inhibition of the JNK signaling pathway [95,101,102].

In our study H_2_S improved motor coordination and fine motor control in hHcy rats which is consistent with findings of anti-inflammation and anti-oxidative effects of H_2_S in animal and cellular models of Parkinson’s disease (PD) [13,85]. Moreover, CBS overexpression in the substantia nigra or striatum ameliorated motor deficits and dopaminergic neuron loss in MPTP (1-methy-4-phenyl-1,2,3,6-tetrahydropyridine)-treated mice or 6-hydroxydopamine (6-OHDA)-lesioned PD rats [103,104]. In skeletal muscle H_2_S exerted beneficial effects by reversing oxidative/ER stress and improving muscle fatigability in CBS^+/−^ mice [67]. At the level of neuromuscular junctions H_2_S increased spontaneous and evoked transmitter release facilitating synaptic transmission [105].

Finally, H_2_S improved learning and memory performance in rats with hHcy which may be explained by reduced oxidative stress, neuroinflammation and apoptosis [85], upregulation of glutathione and aldehyde-dehydrogenase 2 [35,82]. In severe Alzheimer’s disease transgenic mice H_2_S significantly protected against learning and memory dysfunctions and reduced the size of β-Amyloid plaques in the cortex and hippocampus [106]. 

## 5. Conclusions

Taken together, our data suggest that exposure of female rats to high level of homocysteine during pregnancy results in impaired behavior of the offspring in early and late postnatal life, including increased anxiety, decreased muscle strength, altered motor coordination, and weak cognitive performance. Along with oxidative stress we found a decreased concentration of H_2_S and down-regulation of CBS in brain tissues suggesting a role of H_2_S deficit in homocysteine-induced pathologies. Administration of the H_2_S donor, NaHS, to females with hHcy during pregnancy prevented behavioral and biochemical alterations of their offspring suggesting that H_2_S has an important therapeutic potential in the treatment of hHcy-associated disorders. 

## Supplementary Materials

The following are available online at https://www.mdpi.com/2218-273X/10/7/995/s1, Figure S1: Swimming trajectories of rats from control, Hcy, NaHS, and HcyNaHS groups. Table S1: Parameters (time, distance, and speed) swimming of rats from control, Hcy, NaHS, and HcyNaHS groups in water Morris maze learning and probe trials.
