# Supplementary material for: Hydrogen Sulfide Alleviates Anxiety, Motor, and Cognitive Dysfunctions in Rats with Maternal Hyperhomocysteinemia via Mitigation of Oxidative Stress
Source: Biomolecules. 2020 Jul 2;10(7):995. doi: 10.3390/biom10070995 (PMC7408246; doi:10.3390/biom10070995)
Supplement: Supplementary file 1 [file biomolecules-10-00995-s001.pdf]

biomolecules

MDPI

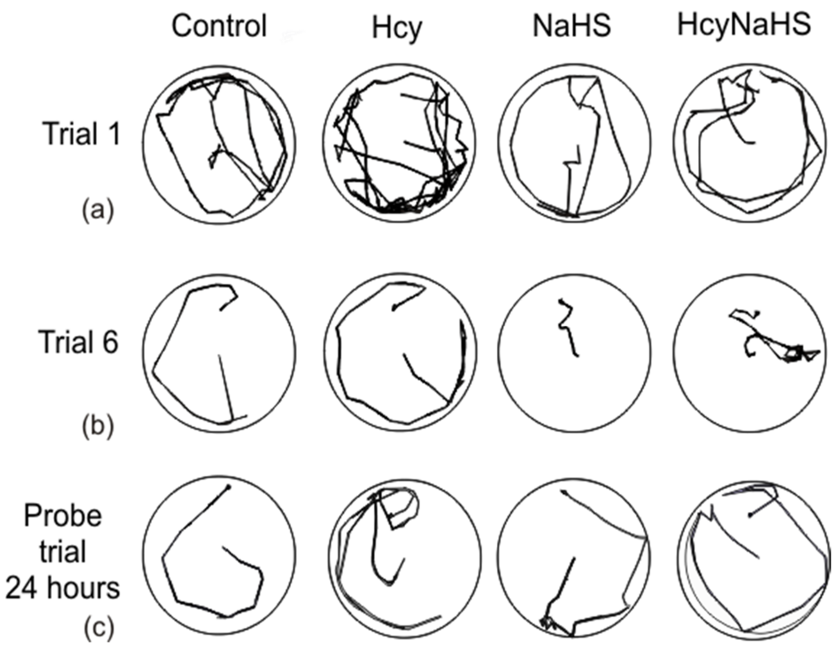

Figure S1. Swimming trajectories of rats from control, Hcy, NaHS, and HcyNaHS groups. Representative swimming paths during the learning trials and probe 24 h trial of rats from control, Hcy, NaHS, and HcyNaHS groups. (**a**) The 1st learning trial thigmotaxis strategy where animal spends most of the time next to the wall was preferred by most rats of all groups. Rats from control and NaHS groups also exhibited target scanning strategy, where they searched in the central part of the arena. Rats from Hcy and HcyNaHS groups used also incursion strategy, where the animal still touches the wall but starts moving inwards. (**b**) In the 6th learning trial the main strategy used by rats from control, NaHS, and HcyNaHS groups was scanning, whereas rats from the Hcy group still used thigmotaxis strategy. (**c**) During 24 h probe trial scanning and target scanning strategies were used by the animals from the control group. Rats from the Hcy group were swimming along the walls and used more simple exploration strategies such as incursion and thigmotaxis. Searching strategies to reach the target quadrant of NaHS and HcyNaHS groups were scanning and incursion.

Table S1. Parameters (time, distance, and speed) of swimming of rats from control, Hcy, NaHS, and HcyNaHS groups in water Morris maze learning and probe trials.

	**Control**	**Hcy**	**NaHS**	**HcyNaHS**
		**Trial 1**		
**Time, s**	30.5 ± 1.8	39.2 ± 1.8*	30.5 ± 2.4#	30.3 ± 2.5#
**Distance, cm**	201.8 ± 25.7	299.4 ± 18.1*	198.1 ± 6.0#	210.9 ± 25.3
**Speed,cm/s**	7.0 ± 1.2	7.0 ± 0.6	6.8 ± 0.6	6.9 ± 0.3
		**Trial 4**		
**Time, s**	11.0 ± 1.5	17.0 ± 1.5*	10.2 ± 1.2#	10.7 ± 1.4#
**Distance, cm**	91.0 ± 11.0	139.4 ± 9.1*	81.0 ± 6.1#	90.0 ± 8.2#
**Speed, cm/s**	7.2 ± 1.4	8.0 ± 0.6	8.0 ± 0.9	8.5 ± 0.6
		**Trial 6**		
**Time, s**	6.2 ± 0.6	9.0 ± 1.1*	5.7 ± 0.5	7.4 ± 0.5
**Distance, cm**	44.2 ± 5.3	65.1 ± 10.1*	44.2 ± 5.1#	51.3 ± 1.4
**Speed, cm/s**	7.2 ± 0.9	7.2 ± 1.0	7.4 ± 0.9	7.2 ± 0.3
		**Probe trial after 1 hr**		
**Time, s**	10.9 ± 0.9	15.6 ± 1.4*	8.3 ± 1.14#	7.9 ± 0.7#
**Distance, cm**	63.3 ± 6.2	91.9 ± 13.1*	46.1 ± 4.5#	45.9 ± 8.8#
**Speed, cm/s**	5.8 ± 0.5	5.8 ± 0.3	5.8 ± 0.6	6.1 ± 1.4
		**Probe trial after 24 hr**		
**Time, s**	14.8 ± 1.6	23.2 ± 2.2*	15.4 ± 1.9	17.4 ± 1.9
**Distance, cm**	92.3 ± 12.1	142.8 ± 12.4*	104.1 ± 14.6	120.4 ± 8.9
**Speed, cm/s**	6.8 ± 1.3	7.7 ± 0.5	7.3 ± 0.9	6.9 ± 0.4

* $p < 0.05$ compared to the control group, # $p < 0.05$ compared to Hcy group.
